# Cellular Immune Response after Vaccination with an Adjuvanted, Recombinant Zoster Vaccine in Allogeneic Hematopoietic Stem Cell Transplant Recipients

**DOI:** 10.3390/vaccines10050809

**Published:** 2022-05-20

**Authors:** Michael Koldehoff, Peter A. Horn, Monika Lindemann

**Affiliations:** 1 Department of Hematology and Stem Cell Transplantation, University Hospital Essen, University Duisburg-Essen, 45147 Essen, Germany; michael.koldehoff@uk-essen.de; 2 Department of Hygiene and Environmental Medicine, University Hospital Essen, University Duisburg-Essen, 45147 Essen, Germany; 3 Institute for Transfusion Medicine, University Hospital Essen, University Duisburg-Essen, 45147 Essen, Germany; peter.horn@uk-essen.de

**Keywords:** varicella-zoster virus, vaccination, ELISpot, hematopoietic stem cell transplantation, shingles, sex-dependency

## Abstract

Hematopoietic stem cell transplant (HSCT) recipients have a high risk of developing primary varicella-zoster virus (VZV) infection and reactivation. VZV vaccination may prevent infection and reactivation. In the current study, recipients of allogeneic HSCT (34 females, 45 males) were vaccinated with adjuvanted, recombinant zoster vaccine Shingrix™, which contains the VZV glycoprotein E. Cellular immunity against various VZV antigens was analyzed by interferon-gamma ELISpot. Peripheral blood mononuclear cells (PBMC) of recipients with versus without prior shingles (*n =* 36 and *n =* 43, respectively) showed approximately twofold higher VZV-specific responses prior to and post vaccination. After the first and second vaccination, ELISpot responses towards the glycoprotein E were significantly higher in males versus females (median of spots increment 18 versus 1 and 17 versus 4, respectively, *p* ≤ 0.02 each). Multivariate analysis showed that shingles and sex both impacts significantly on VZV immunity. Whereas vaccination-induced changes could hardly be detected after stimulation with a whole VZV antigen, there was a significant increase in responses towards glycoprotein E after vaccination (*p* < 0.005). These data indicate that vaccination with Shingrix™ augmented cellular, VZV-specific immunity in HSCT recipients. Shingles and male sex could both be identified as factors leading to increased immunity.

## 1. Introduction

Varicella-zoster virus (VZV), a double-stranded DNA virus, is a member of the herpesvirus family, which causes varicella/chickenpox after primary infection and zoster/shingles after reactivation. Following centripetal axonal transport, circular viral DNA persists in neurons of dorsal root and cranial nerve ganglia, where it can remain quiescent for decades [1]. As all herpesviruses, VZV may reactivate. The cumulative incidence of VZV reactivations leading to shingles increases significantly in older and immunocompromised individuals, as the waning of VZV-specific cellular immunity is an important factor for VZV reactivation [2,3]. The age-dependent increase in shingles is correlated with the decrease in specific T cell immunity [4]. VZV causes a segmental vesicular exanthema in the area of one to three adjoining dermatomes, where it can lead to burning pains and postherpetic neuralgia [1,5]. In the initial phase after hematopoietic stem cell transplantation (HSCT), impaired antiviral T cell function is a major problem and herpesviruses cannot be controlled properly [6]. In these immunocompromised patients, VZV primary infection and reactivation can lead to neurological manifestations such as perivascular demyelination of the central nervous system and encephalitis, hemorrhagic and necrotic skin changes, bacterial superinfections, dissemination of infection, interstitial pneumonia, and other severe organ manifestations [5,7]. After allogeneic HSCT 41% developed VZV reactivation at a median of 227 days post-transplantation [8]. A total of 12% of VZV reactivations occurred in the first 100 days and 88% within the first 24 months [8]. A total of 45% showed disseminated disease, a condition which is lethal in 10% [5].

If varicella or shingles occur after allogeneic HSCT, passive immunization with anti-VZV hyperimmunoglobin (Ig) within 96 h of exposure is recommended [9]. Antiviral therapy with acyclovir or valacyclovir is considered safe and should be given for about one year or longer. Recent data suggest that VZV prophylaxis with acyclovir/valacyclovir for two years, followed by vaccination, results in a lower incidence of VZV disease and may reduce post-herpetic neuralgia [10].

In Germany, the United States and many other countries, a live attenuated vaccine is licensed and its use is recommended for vaccination against primary infection [11,12]. Moreover, to prevent reactivation, the use of a recombinant, adjuvanted VZV glycoprotein E (Shingrix^TM^, GlaxoSmithKline Biologicals S.A., Rixensart, Belgien) is recommended, especially from the age of 60 and for individuals with immunodeficiency [11]. It contains an adjuvant based on liposomes, which serves as an amplifier of immunity [1]. Previous data indicate that vaccination with Shingrix^TM^ could reduce the risk of contracting shingles during lifetime in the general population from 33% to 3% [13]. Moreover, data in kidney transplant recipients indicate it is also effective and may cut the rate of shingles by about half [14]. Considering 130 patients who received Shingrix^TM^ and 130 who received placebo, this study reported a lower rate of suspected cases of shingles in vaccinees (3 vs. 7 suspected cases). Currently, there is no data if vaccination with Shingrix^TM^ could induce specific cellular immunity in allogeneic HSCT recipients, which was addressed in the present study.

We report on a HSCT cohort vaccinated twice with Shingrix^TM^, in which T cell immunity against VZV-specific antigens was analyzed. We stimulated the cells with inactivated whole virus, native glycoprotein and peptides of glycoprotein E (gE), the most abundant and immune-dominant glycoprotein of VZV [15]. Because T cell responses are usually low in HSCT recipients, we decided to use a highly sensitive method, the interferon (IFN)-γ ELISpot assay, which detects specific T cells on a single cell level [16]. Moreover, we analyzed if covariates, such as prior shingles, sex, age, underlying disease, graft-vs.-host disease (GvHD), drug regimen, stem cell source or interval between HSCT, vaccination and testing, had an impact on VZV-specific immunity.

## 2. Materials and Methods

### 2.1. Volunteers

Our study includes 79 HSCT patients tested prior to and post vaccination with Shingrix^TM^ (Table 1). All patients were recruited at the University Hospital Essen (Germany) when they presented for regular follow-up. Enrollment in the study—which comprised the monitoring of VZV (gE)-specific cellular immunity—was offered to all patients in our outpatient clinic who presented to one physician (M.K.) if they were at least six months away from transplantation and at least two months from shingles. They were included between March 2019 and September 2021 if they agreed to participate in the study. Blood samples were drawn prior to vaccination and after the first and second vaccination, together with samples for routine follow-up. However, due to the COVID-19 pandemic, patients did not present regularly at the site and consultation was often by telephone. The group contains 34 females and 45 males, and their median age at the time of first blood sampling was 60 years (range 28–76). A total of 55 out of 79 patients (70%) received a myeloablative preparative regimen and 54 out of 79 (68%) were treated with anti-thymocyte globulin. A total of 8 patients were grafted with bone marrow and 71 with peripheral blood stem cells. Prior to sampling, 36 patients suffered from shingles and 43 did not. The median interval between transplantation and first vaccination was 37 months, between the 2 vaccinations 3.5 months, between first vaccination and blood sampling 2.3 months and between second vaccination and blood sampling 2.6 months.

Altogether, we analyzed 236 samples. Patients were tested up to 3 times before vaccination (85 samples), up to three times after the first vaccination (51 samples) and up to 6 times after the second vaccination (100 samples). We considered all these samples to correlate ELISpot responses to the different VZV antigens. Sequential analyses were performed in 54 patients. It should be noted that the time courses presented in the manuscript only consider the time point closest to vaccination, i.e., one value before vaccination and one after the first and second vaccination, respectively.

For comparison, we performed sequential analyses in four age-matched, healthy controls (median age 62 years; range from 60–65; three males and one female).

The study was conducted according to the guidelines of the Declaration of Helsinki and approved by the Ethics Committee of the University Hospital Essen, Germany (19-9040-BO). Informed consent was obtained from all subjects involved in the study.

### 2.2. Vaccine and Vaccination

The subunit vaccine Shingrix^TM^ contains 50 µg of the adjuvanted, recombinant VZV gE antigen, produced in immortalized ovarian cells of the Chinese hamster (CHO cells) [17]. It is adjuvanted with AS01B containing 50 µg of the plant extract *Quillaja saponaria* Molina, fraction 21 (QS-21) and 50 µg 3-O-desacyl-4′-monophosphoryl lipid A (MPL) from *Salmonella minnesota*. Shingrix^TM^ is licensed for the prevention of shingles and postherpetic neuralgia in adults ≥50 years of age [17]. Vaccination consisted of two 0.5-mL doses, injected into the deltoid muscle. Vaccination was tolerated well and no serious side-effects, e.g., GvHD, occurred.

### 2.3. ELISpot Assay

A total of 9 milliliters of heparinized blood was collected, and peripheral blood mononuclear cells (PBMC) were separated within 24 h after collection by Ficoll gradient centrifugation. Numbers of PBMC were determined by an automated hematology analyzer (XP-300, Sysmex, Norderstett, Germany). To assess VZV-specific cellular immunity, we performed IFN-γ ELISpot assays, using freshly isolated PBMC and two protein antigens and a peptide pool as stimuli. In parallel experiments, we applied a whole VZV antigen (ORYP05, Behring, Marburg, Germany), a native VZV glycoprotein (0.1–10 µg/mL, SERION) and a gE peptide pool (0.5–1 µg/mL per peptide, JPT Peptide Technologies, Berlin, Germany). The whole VZV antigen is produced from human tissue cell cultures infected with VZV and is lyophilized after inactivation and addition of a stabilator. One vial of lyophilized whole VZV antigen was diluted in 2 mL or 8 mL of aqua distillata (dilution 1:2 or 1:8) and 10 µL of the diluted antigen was used per cell culture. The gE peptide pool contains 153 peptides derived from a peptide scan (15-mers with 11 aa overlap) through envelope protein (Swiss-Prot ID: P09259) of VZV strain Dumas. The production of IFN-γ was determined using pre-coated ELISpot plates and a standardized detection system (T-Track^®^ ELISpot kit, Mikrogen GmbH, Neuried, Germany; formerly Lophius Biosciences GmbH, Regensburg, Germany). Cultures of 200,000 freshly isolated PBMC were incubated without and with VZV antigens in 150 µL of AIMV medium (Gibco, Grand Island, NE, USA) at 37 °C for 19 h. Stimulation with the T cell mitogen phytohemagglutinin (PHA, 4 µg/mL) served as a positive control. Colorimetric detection of cytokine secreting cells was performed according to the manufacturer’s instructions. Spot numbers were analyzed by an ELISpot plate reader (AID Fluorospot, Autoimmun Diagnostika GmbH, Strassberg, Germany). VZV-specific spots were determined as stimulated minus non-stimulated (background) values (spots increment). Of note, the negative controls reached a median value of 0, a mean of 0.38 spots and a standard deviation of 0.94 spots. The positive control with PHA indicated that all results included in this study were valid (median 417 spots (range 59–>600).

### 2.4. Statistical Analysis

Data were analyzed using GraphPad Prism 8.4.2.679 (GraphPad Prism Software, San Diego, CA, USA) or IBM SPSS Statistics version 25 (Armonk, New York, NY, USA). ELISpot responses towards different VZV antigens, used at various concentrations, were compared by One-way ANOVA, using a mixed effects model and the Holm–Sidak’s multiple comparisons test. Time courses of ELISpot responses in patients with and without shingles were compared by Two-way ANOVA using the Bonferroni post-test. Results prior to and post vaccination were compared by Wilcoxon matched pairs test, data in two or more independent groups by the Mann–Whitney *U*-test or Kruskal–Wallis test, as appropriate. Correlation analyses of numerical variables were performed by the Spearman test (two-tailed). The impact of shingles and sex on ELISpot responses was analyzed by multivariate analysis (multinomial logistic regression). If not otherwise stated, median values are indicated. The results were considered significant at *p* < 0.05.

## 3. Results

### 3.1. ELISpot Responses to Three Different VZV Antigens

**Figure 1:**
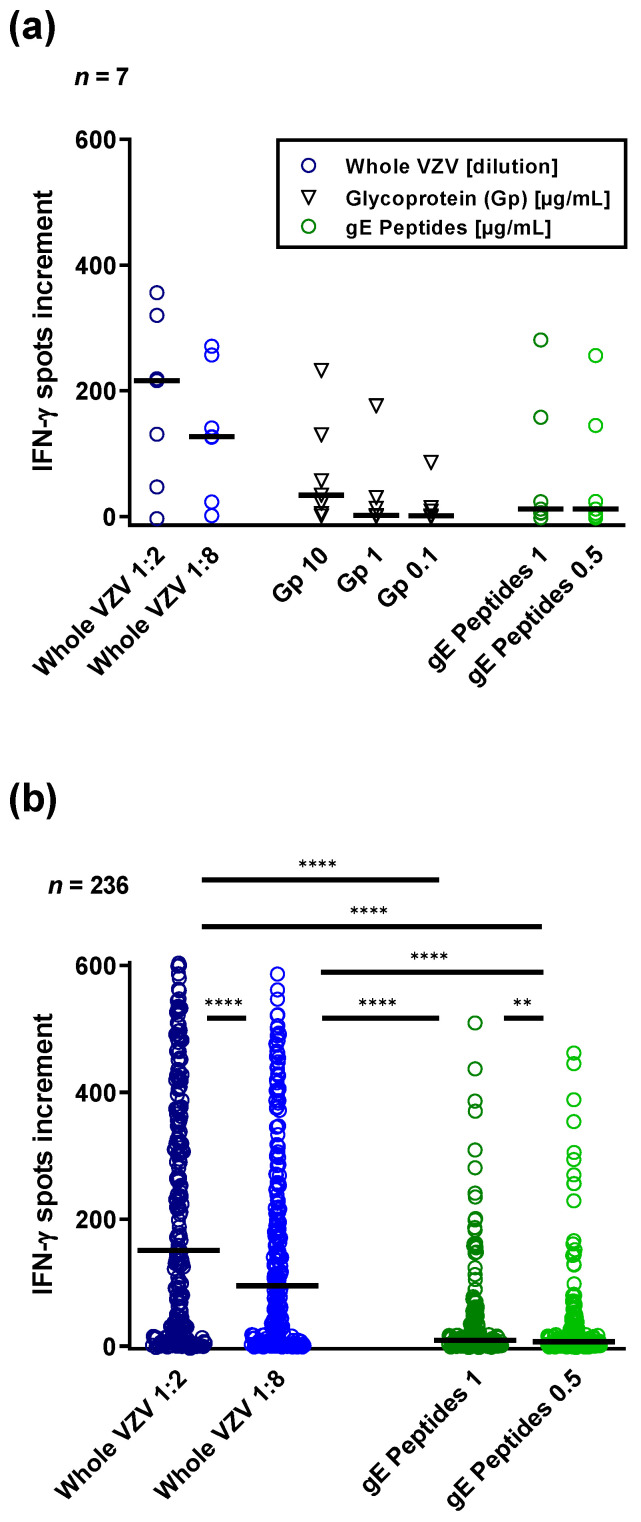
Comparison of ELISpot responses towards various varicella-zoster virus (VZV) antigens in hematopoietic stem cell transplant recipients. We used either an inactivated whole VZV antigen from Behring, a native glycoprotein from SERION (Gp) or a peptide pool of glycoprotein E from JPT (gE peptides). In seven parallel cultures, peripheral blood mononuclear cells (PBMC) of patients vaccinated once or twice with Shingrix™ were grown with these three VZV antigens (**a**). Data on two of the antigens reached similar results in the total cohort, comprising 236 samples prior to and post vaccination with Shingrix™ (**b**). Sampling was performed at a median of 37 months after transplantation. VZV-specific spots were determined as stimulated minus non-stimulated (background) values (spots increment). Horizontal lines represent median values. Data were compared by One-way ANOVA with Holm–Sidak’s multiple comparisons test. ** *p* < 0.01, **** *p* < 0.0001.

In seven HSCT patients vaccinated with Shingrix™ we compared T cell responses towards various VZV antigens, used at different concentrations (Figure 1a). Titration experiments showed a dose-dependency of ELISpot responses in these seven patients and in the total cohort (*n* = 236 samples) (Figure 1a,b). The whole VZV antigen (Behring) induced overall higher ELISpot responses than the native glycoprotein (SERION) and the gE peptide pool (JPT) (Figure 1a). For the subsequent experiments, we have chosen the whole VZV antigen and the gE peptide pool, which is the immunogenic component of the subunit vaccine Shingrix™ (Figure 1b).

### 3.2. Comparison of VZV-Specific Immunity in Stem Cell Transplant Recipients Prior to and Post Vaccination

**Figure 2:**
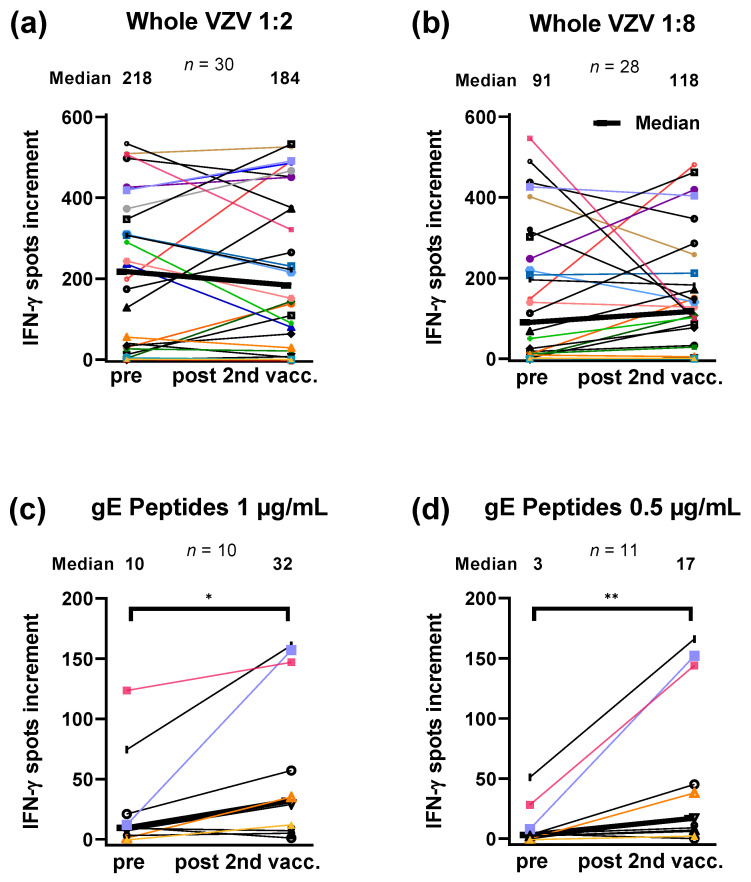
Individual time courses of varicella-zoster virus-specific immunity in hematopoietic stem cell transplant recipients tested prior to and post second vaccination with the adjuvanted, recombinant zoster vaccine Shingrix™. In this cohort, blood sampling was performed at a median of 33 months after transplantation. Vacc-vaccination with Shingrix™; Whole VZV-whole varicella-zoster virus antigen (Behring, dilution 1:2 and 1:8); gE-peptide pool of glycoprotein E (JPT, 1 and 0.5 µg/mL per peptide). The bold black line connects the median values. Paired data were compared by Wilcoxon matched pairs test. * *p* < 0.05, ** *p* < 0.005.

We compared VZV (gE)-specific immunity in HSCT recipients tested prior to and post 2nd vaccination with the adjuvanted, recombinant zoster vaccine Shingrix™ (Figure 2). Of note, this analysis includes only paired data. Whereas responses to the inactivated whole VZV antigen did not differ significantly (Figure 2a,b), responses to the VZV gE peptide were 3.2-fold (1 µg/mL per peptide) or 5.7-fold (0.5 µg/mL per peptide) higher after the second vaccination, reaching *p* values of 0.02 or 0.004, respectively. Moreover, we calculated how many patients showed an at least twofold increase in ELISpot responses, which was defined as criterion for the effectiveness of the vaccination. Considering whole VZV antigen at a dilution of 1:2 or 1:8, 6 out of 30 patients (20%) or 12 out of 28 (43%) fulfilled this criterion. Considering gE peptides at a concentration of 1 and 0.5 µg/mL per peptide, the respective numbers were 6 out of 10 patients (60%) or 10 out of 11 (91%). Thus, changes may be detected with higher sensitivity when lower antigen concentrations were used.

**Figure 3:**
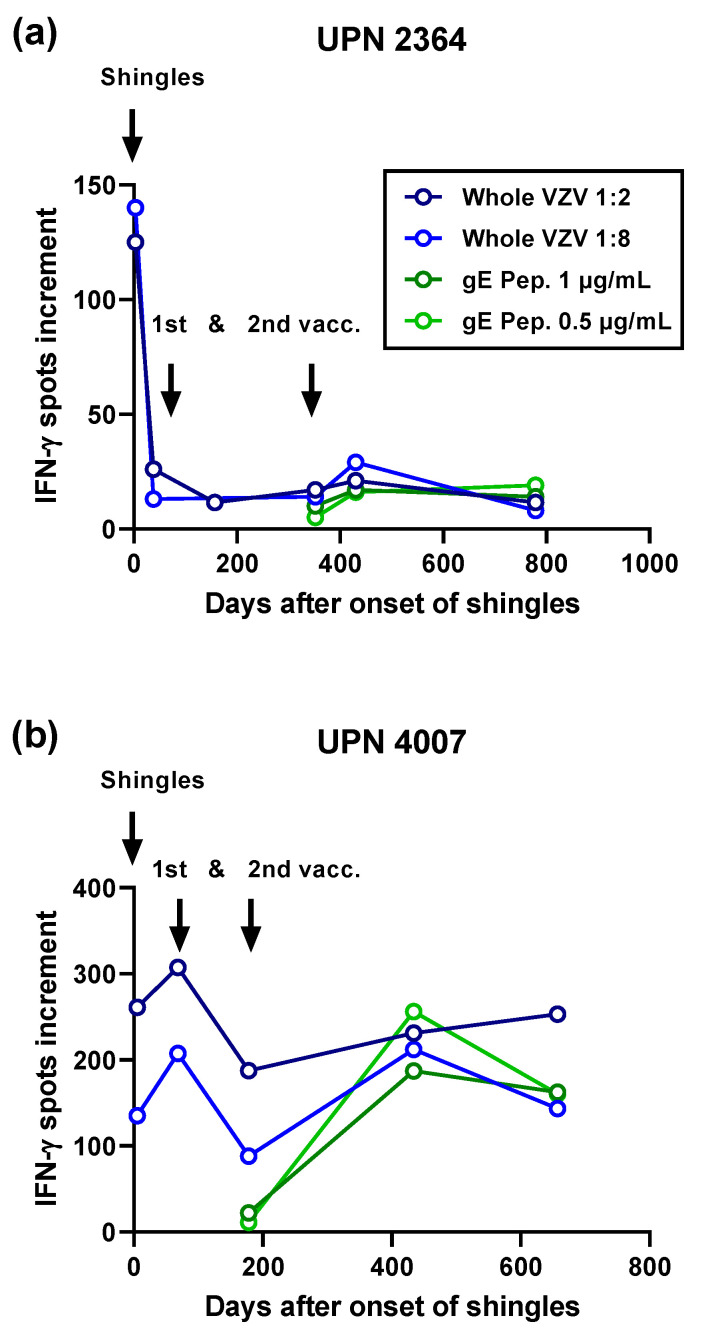
Courses of varicella-zoster virus-specific immunity in two hematopoietic stem cell transplant recipients tested already on day 3 (**a**) and day 5 (**b**) after the onset of shingles and in the follow-up after 1st and 2nd vaccination with the adjuvanted, recombinant zoster vaccine Shingrix™. The patients suffered from shingles 37 months (**a**) and 146 months (12 years) (**b**) after transplantation. Vacc-vaccination with Shingrix™; Whole VZV-whole varicella-zoster virus antigen (Behring, dilution 1:2 and 1:8); gE-peptide pool of glycoprotein E (JPT, 1 and 0.5 µg/mL per peptide).

Two patients could be tested very early after the onset of shingles (Figure 3). In the first patient (UPN 2364), we observed that high T cell responses at day 3 waned substantially within 35 days. Whereas we could not observe an increase in specific immunity after the first vaccination with Shingrix™, the second vaccination induced an increase in responses (Figure 3a). In the second patient (UPN 4007), T cell responses increased from day 5 to day 68 after shingles but decreased after the first vaccination (Figure 3b). Again, we observed an increase after the second vaccination.

**Figure 4:**
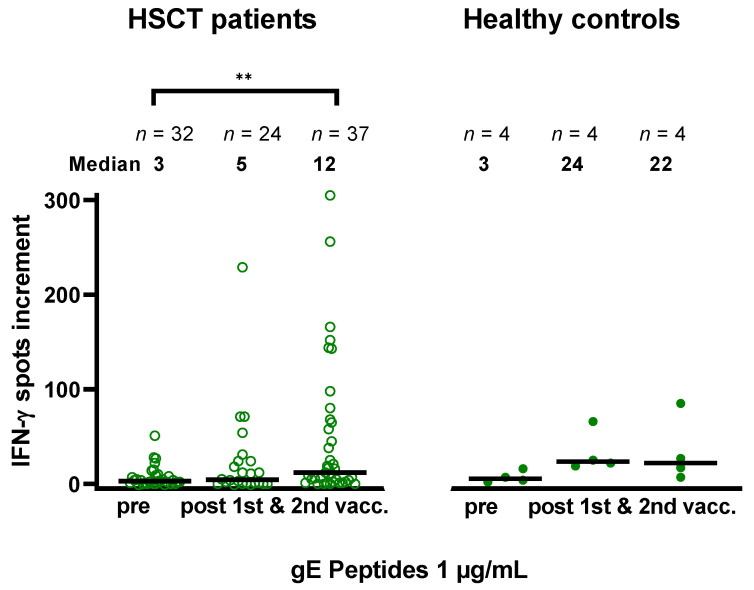
Varicella-zoster virus-specific ELISpot responses in hematopoietic stem cell transplant (HSCT) recipients (open circles) and healthy (filled circles) prior to and after the first and second vaccination with Shingrix™. Cells were stimulated with a peptide pool of glycoprotein E (gE) from JPT, used at concentrations of 1 µg/mL per peptide. Blood sampling in patients was performed at a median of 38 months after transplantation. VZV-specific spots were determined as stimulated minus non-stimulated (background) values (spots increment). Horizontal lines represent median values. Data in patients were compared by one-way ANOVA with Turkey’s multiple comparisons test. ** *p* < 0.005.

Furthermore, considering the first sample of each HSCT patient tested prior to vaccination and after the first and second vaccination, we analyzed if gE-specific ELISpot responses increased after vaccination (Figure 4). Of note, this analysis considered also incomplete data sets, in contrast to the paired data, as shown in Figure 2. Using 1 µg/mL per gE peptide, we could detect a statistically significant increase in cellular responses after the second vaccination in HSCT patients (*p* < 0.005). VZV gE-specific ELISpot responses prior to and post vaccination were compared between HSCT patients and four healthy controls. Prior to vaccination, VZV-specific ELISpot responses towards the gE peptide pool were comparable. However, after the first and second vaccination with Shingrix™, cellular responses were 4.8-fold and 1.8-fold higher in the age-matched healthy controls vs. HSCT patients (median of spots increment 24 vs. 5 and 22 vs. 12, respectively).

**Figure 5:**
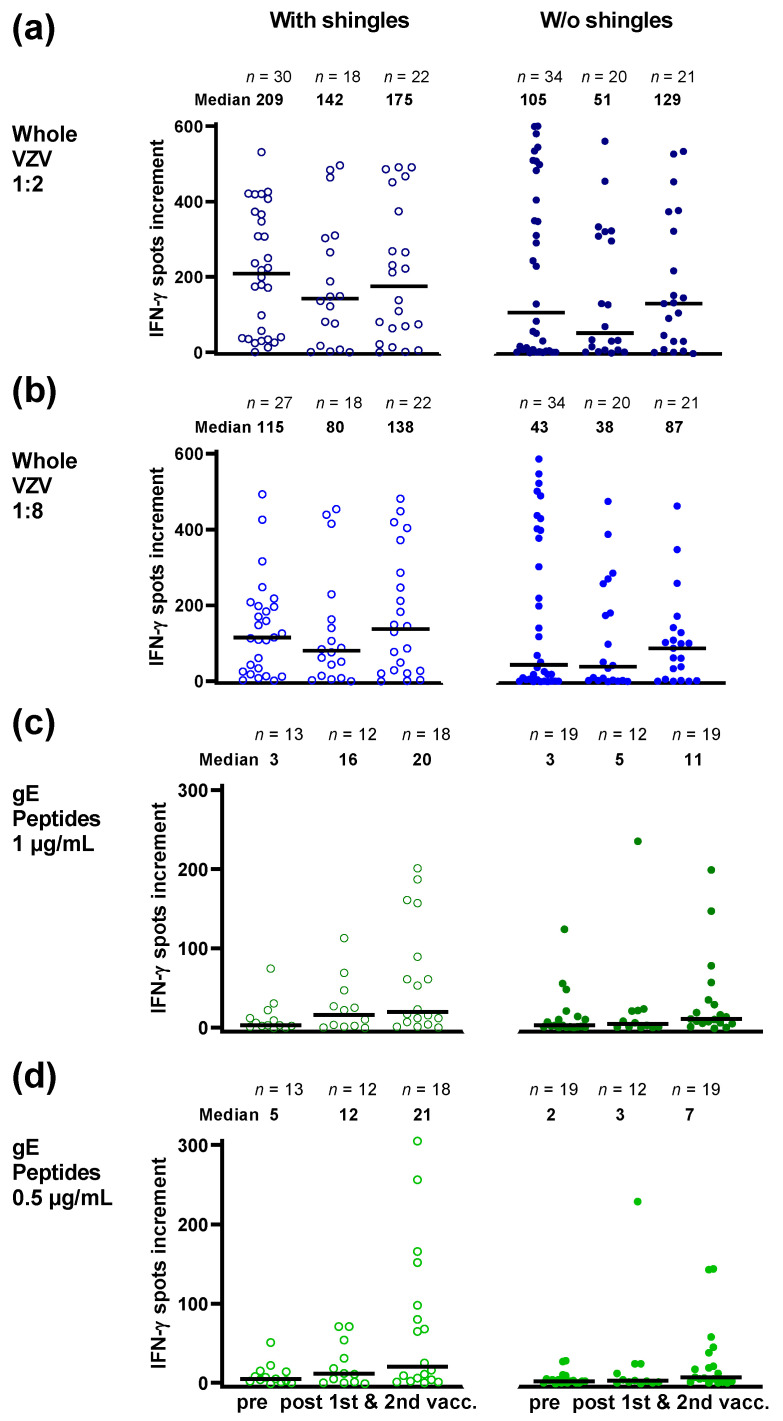
Varicella-zoster virus-specific ELISpot responses in hematopoietic stem cell transplant (HSCT) recipients prior to and after the first and second vaccination with Shingrix™. Panel (**a**) displays responses to a whole VZV antigen (Behring), at a dilution of 1:2, and panel (**b**) at a fourfold higher dilution of 1:8. Panel (**c**) displays responses to a peptide pool of glycoprotein E (gE) from JPT, used at concentration of 1 µg/mL per peptide, in panel (**d**) at 0.5 µg/mL. Open circles (left panel) indicate recipients with shingles and filled circles (right panel) without (w/o) shingles. In patients with and without shingles blood sampling was performed at a median of 35 and 37 months after transplantation, respectively. In patients with and without shingles the median interval between first vaccination and blood sampling was 80 and 61 days and between second vaccination and blood sampling 79 and 78 days, respectively. VZV-specific spots were determined as stimulated minus non-stimulated (background) values (spots increment). Horizontal lines represent median values.

In HSCT patients with vs. without prior shingles, ELISpot responses towards a whole VZV antigen were approximately twofold higher prior to vaccination (209 vs. 105 spots increment for the 1:2 antigen dilution; 115 vs. 43 spots increment for the 1:8 dilution, data represent median values) (Figure 5a,b). Responses in patients with vs. without shingles remained higher after the first and second vaccinations with Shingrix™. However, none of these finding reached statistical significance in univariate analysis. Of note, responses towards the whole VZV antigen prior to and post vaccination did not show a clear increase. However, in patients without prior shingles, responses to the 1:8 dilution of the whole VZV antigen tended to increase.

In contrast, in HSCT patients with vs. without prior shingles, ELISpot responses towards the gE peptides were similar prior to vaccination and responses increased after the first and second vaccination. In patients with prior shingles, the number of spots increment increased 5.3- and 6.7-fold, in patients without prior shingles 1.5- and 3.7-fold (1 µg/mL per peptide) (Figure 5c). Similar results were obtained with 0.5 mg/mL per peptide (with prior shingles: 2.3- and 4.1-fold; without prior shingles: 1.3- and 3.5-fold) (Figure 5d). However, this finding did not reach statistical significance.

**Figure 6:**
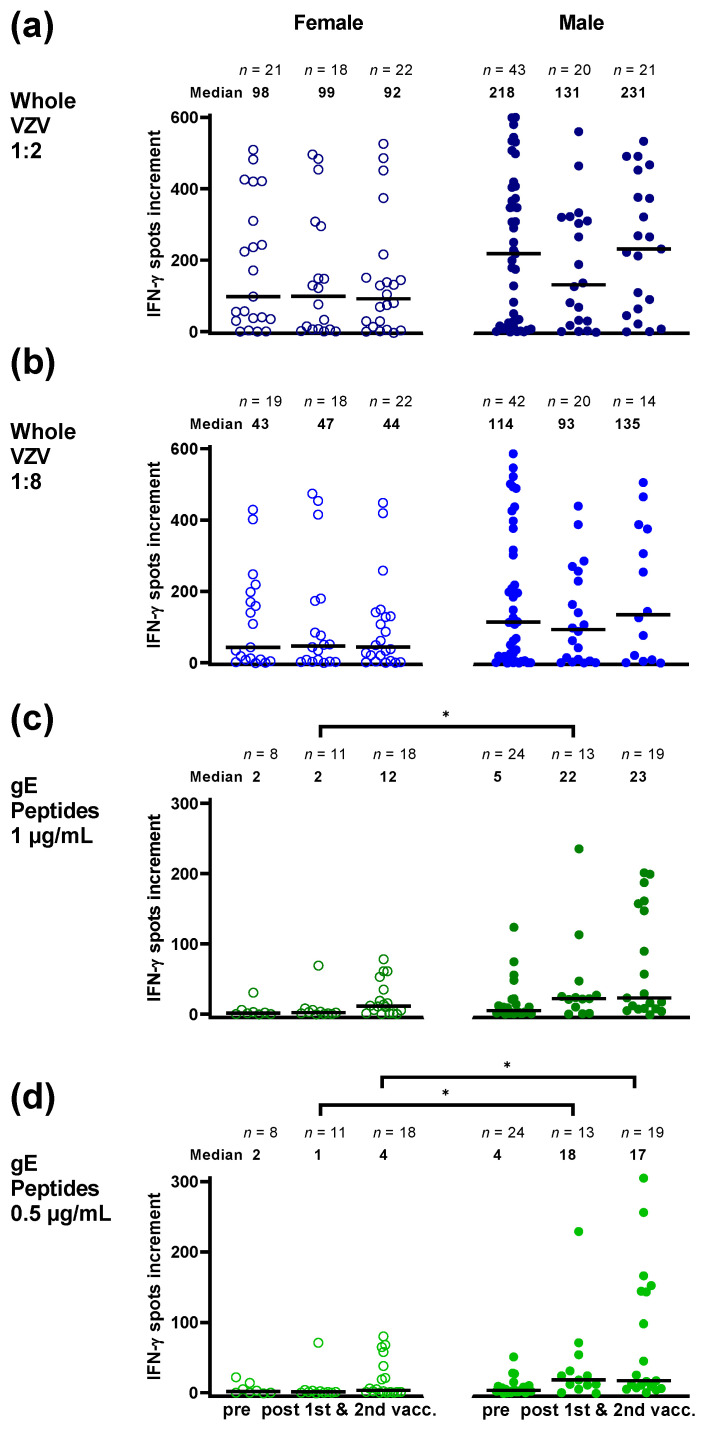
Varicella-zoster virus-specific ELISpot responses in female and male hematopoietic stem cell transplant recipients prior to and after the first and second vaccination with Shingrix™. Panel (**a**) displays responses to a whole VZV antigen (Behring), used at a dilution of 1:2, and panel (**b**) at a dilution of 1:8. Panel (**c**) displays responses to a peptide pool of glycoprotein E (gE) from JPT, used at concentration of 1 µg/mL per peptide, in panel (**d**) at 0.5 µg/mL. Open circles indicate females and filled circles males. In females and males blood sampling was performed at a median of 33 and 39 months after transplantation, respectively. In females the median interval between first vaccination and blood sampling was 53 days, and between second vaccination and blood sampling it was 84 days. In males, the respective intervals were 80 and 78 days. VZV-specific spots were determined as stimulated minus non-stimulated (background) values (spots increment). Horizontal lines represent median values. Data were compared by Mann–Whitney test (* *p* < 0.05).

We investigated whether other factors besides shingles influence VZV-specific cellular immunity. Patient sex had an impact on specific immunity, which was non-significant for the whole VZV antigen (Figure 6a,b), but reached statistical significance for responses to the gE peptide pool after the first and second vaccination (*p* ≤ 0.02) (Figure 6c,d). After the first vaccination, females vs. males showed median numbers of spots increment of 2 vs. 22 (1 µg/mL per peptide, *p* = 0.02) (Figure 6c) and 1 vs. 18 (0.5 µg/mL per peptide, *p* = 0.01) (Figure 6d), respectively. After the second vaccination, the corresponding spot numbers were 12 vs. 23 (*p* = 0.07) and 4 vs. 17 (*p* = 0.02) (Figure 6c,d).

However, ELISpot responses did not correlate significantly with age, underlying disease, acute or chronic GvHD, myeloablative preparative regimen, treatment with anti-thymocyte globulin or stem cell source (bone marrow vs. peripheral blood stem cells). Moreover, we could not observe a significant correlation with the interval between transplantation and vaccination, the interval between first and second vaccination, and the interval between first or second vaccination and blood sampling, respectively.

The impact of shingles and sex on ELISpot responses was then examined by multivariate analysis (Table 2). Findings of the univariate analyses could be confirmed, i.e., shingles and sex could be identified as independent factors influencing cellular VZV immunity. For example, responses to 1 µg/mL gE peptides after the first vaccination correlated significantly with prior shingles (*p* = 0.03) and sex (*p* = 0.04).

### 3.3. Correlation of VZV-Specific Immunity Measured with Various VZV Antigens and at Various Time Points

Spearman analysis indicated that ELISpot responses towards the peptide pool of gE and the other VZV antigens were positively correlated in the majority of cases. As a first step, we analyzed three VZV antigens, used at different concentrations in seven HSCT patients (Table 3). Correlation between responses to gE and the whole VZV antigen was weak (*r =* 0.36–0.54).

We then analyzed the total cohort (*n* = 236 samples). Correlation coefficients were similar, and all correlations were highly significant (*p* < 0.0001) (Table 4). As expected, responses towards the same antigen, used at different concentrations, showed a stronger correlation (*r* = 0.89–0.96, *p* < 0.0001) than responses towards various VZV antigens (*r* = 0.44–0.46, *p* < 0.0001). Moreover, correlations between responses to gE and whole VZV antigen were stronger after first and second vaccination than prior to vaccination.

In addition, we performed Spearman analyses for ELISpot results obtained at different time points. The respective correlation analyses showed moderate to strong correlation (*r =* 0.68–0.91). (Table 5). ELISpot responses prior to vaccination were thus predictive of responses after the first and after the second vaccination. Moreover, responses prior to the first and second vaccination correlated significantly (*p* < 0.005).

## 4. Discussion

The current study indicates that vaccination with Shingrix^TM^ augmented cellular immunity in allogeneic HSCT recipients, which had not yet been shown. A comparative analysis of various VZV antigens showed that gE may be more suitable than whole VZV antigens for measuring vaccine-induced VZV gE-specific immunity. We observed that cellular responses towards gE increased significantly after vaccination, whereas responses to whole VZV did not. gE is the most abundant and immune-dominant glycoprotein expressed on the surface of VZV-infected cells [15] and constitutes a major target for VZV-specific antibody response [18]. As compared to gE, the whole VZV antigen contains additional T cell epitopes, which most likely contributes to the overall more than 5-fold higher spot numbers after the second vaccination. When stimulating cells with whole VZV antigen presumably the proportion of the cells responding to gE after vaccination was too low to be detectable. Previously, a strong correlation of glycoprotein-specific antibodies and protection against varicella has been shown [19]. Moreover, ELISA data indicate that IgG antibodies against whole VZV and against the gE show good correlation, when analyzing the data qualitatively (positive/negative, 99% agreement) [20] and quantitatively (correlation coefficient of 0.86%) [21]. Similar to these antibody data, we observed a significant correlation of cellular responses to whole VZV antigens and gE. Previous data by Cassaniti et al. indicate that the ELISpot after stimulation with gE peptides is mainly a CD4 T cell response [22]. This group measured immunity in (unvaccinated) kidney transplant recipients and found a lower level of ELISpot responses than we observed in the current study. Most likely, various immunosuppressive treatments are a major factor causing this difference.

In a cohort of 32 kidney transplant recipients, there are already data on T cell immunity after vaccination with Shingrix™ [14]. Immunity was determined by intracellular cytokine staining and detection by flow cytometry, after stimulation of CD4 T cells with a pool of peptides covering the gE ectodomain. This study showed a vaccine response rate for cell-mediated immunity of 71% at month 2, defined as an at least twofold increase in responses after two vaccinations. Moreover, considering 130 patients who received Shingrix™ and 130 who received placebo, a lower rate of suspected shingles was reported in Shingrix™ vs. placebo recipients (3 vs. 7 suspected cases) [14]. Shingrix™ vaccination had no effect on allograft function, as defined by serum creatinine or rejection rate. The incidence of shingles was up to nine times higher in immunosuppressed solid organ transplant recipients than in the general population [14].

In autologous HSCT recipients ≥18 years of age, a vaccination efficacy of 68% has been reported, summarizing results of three studies [14]. However, only 3 out of 17 allogeneic stem cell transplant recipients (18%) showed detectable antibody responses after vaccination with two doses of Shingrix™ [23]. To increase VZV immunity in these patients, vaccination of the respective donors could be considered. Previously, we showed that antimicrobial immune responses can be transferred via HSCT, which then protects the donor and recipient [24]. However, after a single dose of a live attenuated VZV vaccine, 18 of the 31 pediatric HSCT recipients (58%) were seropositive [25]. Moreover, after vaccination with a live attenuated VZV vaccine 19 HSCT recipients (autologous or allogeneic, older than 50 years) showed a significant increase in ELISpot responses [26]. In this study, a UV-inactivated VZV preparation of VZV antigens derived from clarified cell culture supernatants from VZV-infected MRC-5 cells was used to stimulate cell cultures [27]. Similar to our study, the authors [26] described that patients with vs. without a history of shingles tended to have higher ELISpot responses at baseline. In detail, ELISpot responses to whole VZV antigen were 1.7-fold higher in patients with shingles tested 2–5 years after HSCT by Chun et al. [26]. In the current study responses to the whole VZV antigen were 2.0- to 2.7-fold higher at baseline, depending on the antigen concentration, as detailed in Figure 5. Moreover, after vaccination responses to the whole VZV antigen were up to 2.8-fold and to gE peptides up to 4.0-fold higher in patients with shingles, respectively. The current study is not suitable to define if vaccination in allogeneic HSCT recipients was protective, because this warrants a larger study cohort and a placebo control group. However, a previous study on allogeneic HSCT recipients determined the rate of shingles following two doses of Shingrix™ [28]. The authors described that 3 out of 150 patients suffered from shingles (28.3/1000 person-years); which appears as low as compared to 41% of VZV reactivations at a median of 227 days (range 45–346 days) post-transplantation [8].

Our study has several limitations. The major point is that there was variation in the timing of vaccinations and blood sampling. The patients were not called in especially for the study and most of them presented at our site only rarely due to the COVID-19 pandemic. However, there was no special evaluation scheme depending on subgroups. Moreover, a shortage of the vaccine may have extended the interval between the two vaccinations. To partly address this point, we presented a subset of data separately (Figure 2). We showed paired data prior to vaccination and after the second vaccination, which even more clearly show the increase in ELISpot response to gE peptides after the second vaccination. In addition, our study is rather small. Nevertheless, it comprises patients of only one transplant center. Thus, the treatment was more homogeneous than in a multicenter study.

In the current study on adult allogeneic HSCT recipients, we show that cellular immunity against gE of VZV increased after vaccination with Shingrix™. Furthermore, we identify prior shingles and male sex as factors leading to increased VZV-specific immunity. Whereas the influence of prior shingles was expected at first glance, the influence of sex is less clear. VZV reactivation leads to an in vivo expansion of VZV-specific T cells, which may be further “boostered” after vaccination. Previous literature indicates that immune responses in females and males may differ, for example the concentration of cytokines or vaccine antibodies [29,30,31,32,33,34,35,36]. In females, cytomegalovirus pp65-specific IL-21 ELISpot responses were higher [33] or antibody titers after vaccination against hepatitis B or SARS-CoV-2 virus were increased [29,37]. It thus appears possible that also VZV-specific immunity shows a sex dependency.

## 5. Conclusions

In allogeneic HSCT recipients, vaccination with the adjuvanted, recombinant vaccine Shingrix™, which contains the VZV gE, led to a significant increase in cellular in vitro responses towards VZV gE. As compared to the controls, cellular responses were weaker. The current data indicate that patients with prior shingles and males exhibit higher VZV-specific IFN-γ responses. It needs to be clarified whether the higher ELISpot responses correlate with better protection against VZV infection and reactivation.

## Figures and Tables

**Table 1:** Characteristics of 79 hematopoietic stem cell transplant recipients tested prior to and post vaccination with Shingrix™.

Variable	Group	Absolute Number or Median (Range)
Sex	Female	34
	Male	45
Age (years)		60 (28–76)
Underlying disease	Acute leukemia	33
	Myelodysplastic syndrome	14
	Myeloproliferative neoplasia	18
	Lymphoma	11
	Other/not specified	2
Acute GvHD	0	4
	I	27
	II	46
	III	1
	IV	1
Chronic GvHD	None	4
	Limited	56
	Extensive	19
Interval transplantation– 1st vaccination		37 months (8–402)
Interval 1st vaccination– 2nd vaccination		3.5 months (1–16)
Interval vaccination– (first) blood sampling	1st	2.3 months (0.3–23)
	2nd	2.6 months (0.2–13)

GvHD-graft-vs. host disease.

**Table 2:** Multivariate analysis of the impact of shingles and sex on VZV-specific ELISpot responses in patients after hematopoietic stem cell transplantation.

Time Points	Antigen for ELISpot	*n*	*p (Shingles)*	*p (Sex)*
Pre vacc.	VZV 1:2	64	0.01	0.07
	VZV 1:8	61	0.006	0.11
	gE 1	32	0.30	0.69
	gE 0.5	32	0.11	0.09
Post 1st vacc.	VZV 1:2	38	0.04	0.04
	VZV 1:8	38	0.06	0.04
	gE 1	24	0.03	0.04
	gE 0.5	24	0.26	0.09
Post 2nd vacc.	VZV 1:2	43	0.02	0.02
	VZV 1:8	43	0.03	0.04
	gE 1	37	0.06	0.03
	gE 0.5	37	0.07	0.02

Vacc.-vaccination with Shingrix™; VZV-whole varicella-zoster virus antigen (Behring, dilution 1:2 and 1:8); gE-peptide pool of glycoprotein E (JPT, 1 and 0.5 µg/mL per peptide).

**Table 3:** Spearman correlation of ELISpot responses towards a peptide pool of gE (1 µg/mL) and towards various other varicella-zoster virus (VZV) antigens in seven hematopoietic stem cell transplant recipients vaccinated with Shingrix™.

Antigen for ELISpot	Dilution or Concentration	*r*	*p*
Whole VZV Behring	1:2	0.54	0.24
	1:8	0.36	0.44
Glycoprotein SERION	10 µg/mL	−0.07	0.91
	1 µg/mL	0.72	0.08
	0.1 µg/mL	0.67	0.12
Glycoprotein E Peptides JPT	gE peptides 0.5 µg/mL	1.00	0.0004

**Table 4:** Spearman correlation of ELISpot responses towards a whole varicella-zoster virus (VZV) antigen and a peptide pool of glycoprotein E in hematopoietic stem cell transplant recipients prior to and post vaccination with Shingrix™.

All Samples (*n* = 172–236)	Variable 2							
**Variable 1**	VZV 1:2		VZV 1:8		gE 1		gE 0.5	
	***r***	***p*** ****	***r***	***p*** ****	***r***	***p*** ****	***r***	***p*** ****
VZV 1:2	1		0.96		0.44		0.46	
VZV 1:8	0.96		1		0.44		0.46	
gE 1	0.44		0.44		1		0.89	
gE 0.5	0.46		0.46		0.89		1	
**Pre vacc.** (***n* = 32–64**)	**Variable 2**							
**Variable 1**	VZV 1:2		VZV 1:8		gE 1		gE 0.5	
	***r***	***p***	***r***	***p***	***r***	***p***	***r***	***p***
VZV 1:2	1		0.97	<0.0001	0.27	0.13	0.39	0.003
VZV 1:8	0.96	<0.0001	1		0.34	0.06	0.48	0.006
gE 1	0.27	0.13	0.34	0.06	1		0.77	<0.0001
gE 0.5	0.39	0.003	0.48	0.006	0.77	<0.0001	1	
**Post 1st vacc.** (***n* = 24–38**)	**Variable 2**							
**Variable 1**	VZV 1:2		VZV 1:8		gE 1		gE 0.5	
	***r***	***p***	***r***	***p***	***r***	***p***	***r***	***p***
VZV 1:2	1		0.97	<0.0001	0.81	<0.0001	0.59	0.002
VZV 1:8	0.97	<0.0001	1		0.79	<0.0001	0.56	0.005
gE 1	0.81	<0.0001	0.79	<0.0001	1		0.78	<0.0001
gE 0.5	0.59	0.002	0.56	0.005	0.78	<0.0001	1	
**Post 2nd vacc.** (***n* = 37–42**)	**Variable 2**							
**Variable 1**	VZV 1:2		VZV 1:8		gE 1		gE 0.5	
	***r***	***p***	***r***	***p***	***r***	***p***	***r***	***p***
VZV 1:2	1		0.93	<0.0001	0.45	0.005	0.53	0.001
VZV 1:8	0.93	<0.0001	1		0.55	<0.0001	0.61	<0.0001
gE 1	0.45	0.005	0.55	<0.0001	1		0.95	<0.0001
gE 0.5	0.53	0.001	0.61	<0.0001	0.95	<0.0001	1	

VZV-whole varicella-zoster virus antigen (Behring, dilution 1:2 and 1:8); gE-peptide pool of glycoprotein E (JPT, 1 and 0.5 µg/mL per peptide). Please note that all correlations were highly significant when considering all samples (**** *p* < 0.0001).

**Table 5:** Spearman correlation analysis of VZV-specific ELISpot responses prior to and post vaccination in patients after hematopoietic stem cell transplantation.

Time Points	Antigen for ELISpot	*n*	*r*	*p*
Pre–post 1st vacc.	VZV 1:2	30	0.91	<0.0001
	VZV 1:8	27	0.89	<0.0001
	gE 1	7	0.85	0.02
	gE 0.5	7	0.90	0.006
Pre–post 2nd vacc.	VZV 1:2	31	0.81	<0.0001
	VZV 1:8	28	0.71	<0.0001
	gE 1	10	0.68	0.03
	gE 0.5	10	0.76	0.01
Post 1st vacc.–post 2nd vacc.	VZV 1:2	26	0.87	<0.0001
	VZV 1:8	26	0.83	<0.0001
	gE 1	14	0.73	0.003
	gE 0.5	14	0.76	0.002

Vacc.-vaccination with Shingrix™; VZV-whole varicella-zoster virus antigen (Behring, dilution 1:2 and 1:8); gE-peptide pool of glycoprotein E (JPT, 1 and 0.5 µg/mL per peptide).

## Data Availability

The data presented in this study are available on request from the corresponding author. The data are not publicly available due to privacy restrictions.
